# Influence of right ventricular ejection fraction on the occurrence of arrhythmic events in patients with systolic dysfunction

**DOI:** 10.1186/1532-429X-16-S1-O32

**Published:** 2014-01-16

**Authors:** Umjeet S Jolly, Immaculate Nevis, Fahad S Almehmadi, Mohammad Zahrani, Mahmoud Bokhari, John Stirrat, Raymond Yee, James A White

**Affiliations:** 1 Division of Cardiology, London Health Sciences Centre, London, Ontario, Canada; 2 Robarts Research Institute, Western University, London, Ontario, Canada; 3 Departments of Cardiac Sciences and Radiology, University of Calgary, Calgary, Alberta, Canada; 4 Internal Medicine, London Health Sciences Centre, London, Ontario, Canada; 5 Lawson Health Research Insitute, Western University, London, Ontario, Canada

## Background

Left ventricular ejection fraction (LVEF) is the primary risk stratification tool used for therapeutic decision making related to device therapy. However, an influence of right ventricular dysfunction on cardiovascular events is becoming apparent. The incremental prognostic value of right ventricular ejection fraction (RVEF) on future arrhythmic events among those being considered for device therapy has not been well examined. In this study we evaluate the significance of MRI-based measurement of RVEF versus other clinical and MRI-based variables for their ability to predict Sudden Cardiac Death (SCD) or Appropriate ICD therapy.

## Methods

Consecutive patients with cardiomyopathy were evaluated for device candidacy. All patients underwent a standard Late Gadolinium Enhancement (LGE) MRI protocol followed by a blinded core-laboratory based quantification of left ventricle (LV) and right ventricle (RV) volumes, ejection fraction (EF) and total hyperenhancement (HE). Patients were stratified according to the presence or absence of significant RV systolic dysfunction, defined as an RVEF ≤45%. All patients were followed for the occurrence of SCD or appropriate ICD therapy. The secondary outcome was heart failure admission or non-sudden cardiac death. All clinical and MRI-based variables were evaluated for associations with the primary and secondary outcomes with Cox proportional multivariable regression analysis also performed.

## Results

A total of 318 patients (149 ischemic, 169 non-ischemic) were followed over a median of 467 days. At the end of follow-up 49 patients (15.4%) suffered a primary outcome (10 SCD and 39 appropriate therapies). Baseline clinical characteristics were similar among those with and without the primary outcome with the exception of prior history of ventricular arrhythmia and ischemic etiology (p < 0.05). Following adjustment for etiology, LVEF, LVEDV, and Total HE, those with an RVEF≤45% were more likely to experience the primary outcome (HR 2.2; 95% 1.23 to 3.79, p value = 0.007) and the secondary outcome (HR 2.91; 95% CI 1.68 to 5.06, p value = 0.00015) of interest.

## Conclusions

Patients with right ventricular dysfunction, defined as RVEF≤45% by cardiac MRI, are at an increased risk of future arrhythmic events. Similarly, these individuals are at an elevated risk of heart failure admission and non-sudden cardiac death.

## Funding

This research was supported by the Imaging in Cardiovascular Therapeutic grant from the Ontario Research Fund and the Canada Foundation of Innovation.

**Figure 1:**
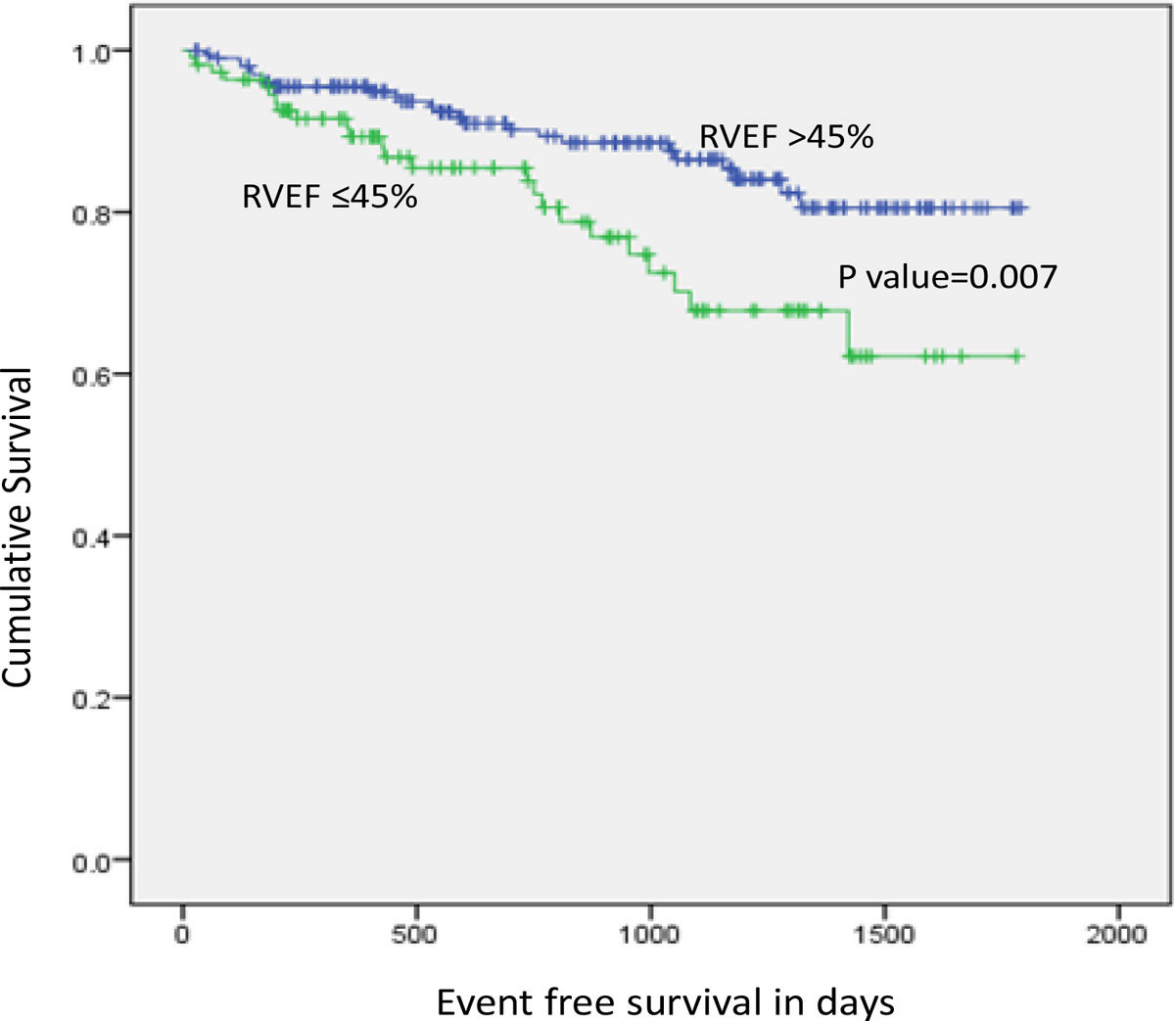
**Kaplan-Meier curves showing the separation between the RVEF > 45% group versus the RVEF < 45% group in event free days survival for the primary outcome**.

